# Characterization of Human Medullary Thyroid Carcinoma Glycosphingolipids Identifies Potential Cancer Markers

**DOI:** 10.3390/ijms221910463

**Published:** 2021-09-28

**Authors:** Karin Säljö, Anders Thornell, Chunsheng Jin, Olov Norlén, Susann Teneberg

**Affiliations:** 1 Department of Plastic Surgery, Institute of Clinical Sciences, Sahlgrenska Academy, University of Gothenburg, S-41345 Gothenburg, Sweden; karin.saljo@vgregion.se; 2 Region Västra Götaland, Sahlgrenska University Hospital, S-41345 Gothenburg, Sweden; anders.thornell@vgregion.se; 3 Department of Surgery, Institute of Clinical Sciences, Sahlgrenska University Hospital, University of Gothenburg, S-41345 Gothenburg, Sweden; 4 Department of Medical Biochemistry and Cell Biology, Institute of Biomedicine, Sahlgrenska Academy, University of Gothenburg, S-40530 Gothenburg, Sweden; chunsheng.jin@medkem.gu.se; 5 Department of Surgical Sciences, Uppsala University, S-75185 Uppsala, Sweden

**Keywords:** human medullary thyroid cancer marker, glycosphingolipid characterization, mass spectrometry, Lewis y antigen, Forssman antigen

## Abstract

Medullary thyroid carcinoma (MTC) accounts for only 1–2% of thyroid cancers; however, metastatic MTC is a mortal disease with no cure. In this study, glycosphingolipids were isolated from human MTCs and characterized by mass spectrometry and binding of carbohydrate recognizing ligands. The tissue distribution of selected compounds was investigated by immunohistochemistry. The amount of acid glycosphingolipids in the MTCs was higher than in the normal thyroid glands. The major acid glycosphingolipid was the GD3 ganglioside. Sulfatide and the gangliosides GM3 and GD1a were also present. The majority of the complex non-acid glycosphingolipids had type 2 (Galβ4GlcNAc) core chains, i.e., the neolactotetraosylceramide, the Le^x^, H type 2 and x_2_ pentaosylceramides, the Le^y^ and A type 2 hexaosylceramides, and the A type 2 heptaosylceramide. There were also compounds with globo (GalαGalβ4Glc) core, i.e., globotriaosylceramide, globotetraosylceramide, the Forssman pentaosylceramide, and the Globo H hexaosylceramide. Immunohistochemistry demonstrated an extensive expression av Le^y^ in the MTC cells and also a variable intensity and prevalence of Globo H and Le^x^. One individual with multiple endocrine neoplasia type 2B expressed the Forssman determinant, which is rarely found in humans. This study of human MTC glycosphingolipids identifies glycans that could serve as potential tumor-specific markers.

## 1. Introduction

Medullary thyroid carcinoma (MTC) accounts for only 1–2% of thyroid cancers [1]. Unlike the rather common follicular cell-derived thyroid cancers, papillary thyroid cancer and follicular thyroid cancer, MTC originates from parafollicular C-cells [2]. Thus, radioiodine imaging and treatment, successfully used for iodine-avid follicular cell-derived cancers, is not applicable to MTC. MTC also has a high propensity to metastasize both to lymph nodes and parenchymal organs rendering the disease difficult to cure with surgery, especially since MTC metastases can be difficult to image with currently available radiological methods. Chemotherapy has little effect in MTC, and available oncological treatments such as tyrosine kinase inhibitors offers partial response in less than 50% of all patients [3,4]. Therefore, a search for novel druggable targets for MTC is warranted.

One hallmark of cancer is aberrant glycosylation that is due to abnormally expressed glycosyltransferases and glycosidases in tumor cells and leads to the generation of tumor-associated carbohydrate antigens (TACAs) [5,6,7,8]. There are several different forms of cancer-associated alterations of cell surface glycoconjugates. TACAs may be due to an enhanced expression of certain carbohydrate structures, or an accumulation of precursor carbohydrate chains as well as appearance of novel carbohydrate structures. TACAs are of interest in the search for anti-cancer immunotherapeutics since they may allow the differentiation between tumor and normal cells.

The changes in protein glycosylation in thyroid cancers has been the subject of many studies (reviewed in [9,10]). However, there are only a few studies about thyroid cancer gangliosides [11,12]. Thus, a thorough characterization of thyroid cancer glycosphingolipids with the methods of today has not been conducted, and the non-acid glycosphingolipids have not been characterized. In this study we have isolated acid and non-acid glycosphingolipids of human MTCs. The glycosphingolipids were characterized by mass spectrometry, enzymatic digestion, and by binding of a battery of carbohydrate recognizing ligands, the tissue distribution of selected compounds was then investigated by immunohistochemistry.

## 2. Results

### 2.1. Isolation of Human Medullary Thyroid Cancer Glycosphingolipids

Total acid and non-acid glycosphingolipid fractions were isolated from pooled human medullary carcinomas of the thyroid by standard procedures [13]. This gave 15.6 mg acid and 4.5 mg non-acid glycosphingolipids/g dry weight tissue (Supplementary Table S1). In our recent study of glycosphingolipids of normal human thyroid gland, 2.0 mg acid and 2.5 mg non-acid glycosphingolipids/g dry weight tissue were obtained [14]. Thus, the amount of acid glycosphingolipids was substantially increased in the medullary thyroid cancers.

**Figure 1:**
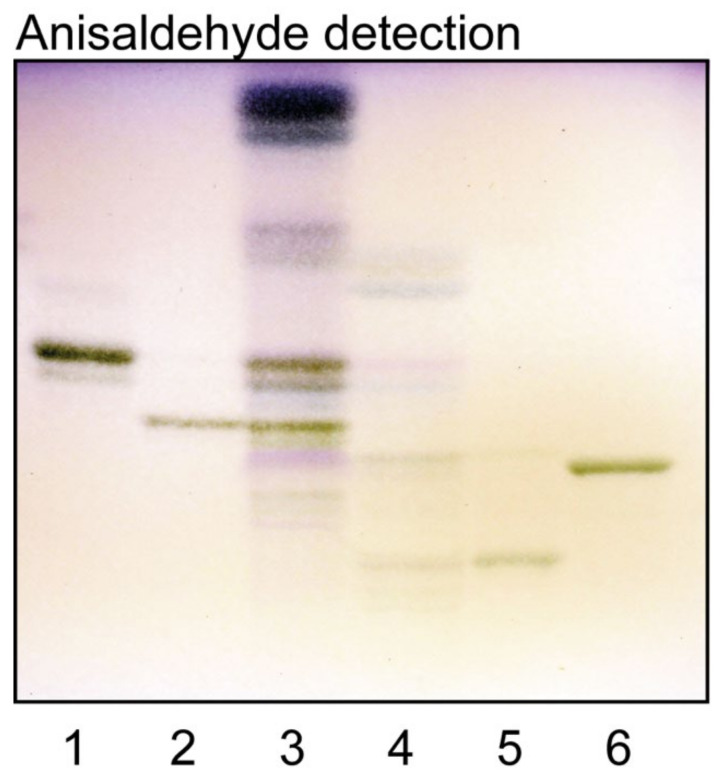
Thin-layer chromatography of the glycosphingolipids isolated from human medullary thyroid cancer. Thin-layer chromatogram after detection with anisaldehyde. Lane 1, reference globotriaosylceramide (Galα4Galβ4Glcβ1Cer), 4 μg; lane 2, reference globotetraosylceramide (GalNAcβ3Galα4Galβ4Glcβ1Cer), 4 μg; lane 3, total non-acid glycosphingolipids isolated from human medullary thyroid cancer. 40 μg; lane 4, total acid glycosphingolipids isolated from human medullary thyroid cancer. 40 μg; lane 5, reference GD3 ganglioside (Neu5Acα8Neu5Acα3Galβ4Glcβ1Cer), 4 μg; lane 6, reference GM3 ganglioside (Neu5Acα3Galβ4Glcβ1Cer), 4 μg.

The total acid fraction is shown in Figure 1, lane 4. The major bands co-migrated with reference GD3 and GM3 gangliosides (Figure 1, lanes 5 and 6), and there was also a fast-migrating band in the sulfatide region. The total non-acid glycosphingolipid fraction (Figure 1, lane 3) had a number of compounds migrating as mono-, di-, tri- tetra- and pentaosylceramides, and also some minor slow-migrating compounds.

### 2.2. Characterization of the Acid Glycosphingolipids from Human Medullary Thyroid Cancer

**Figure 2:**
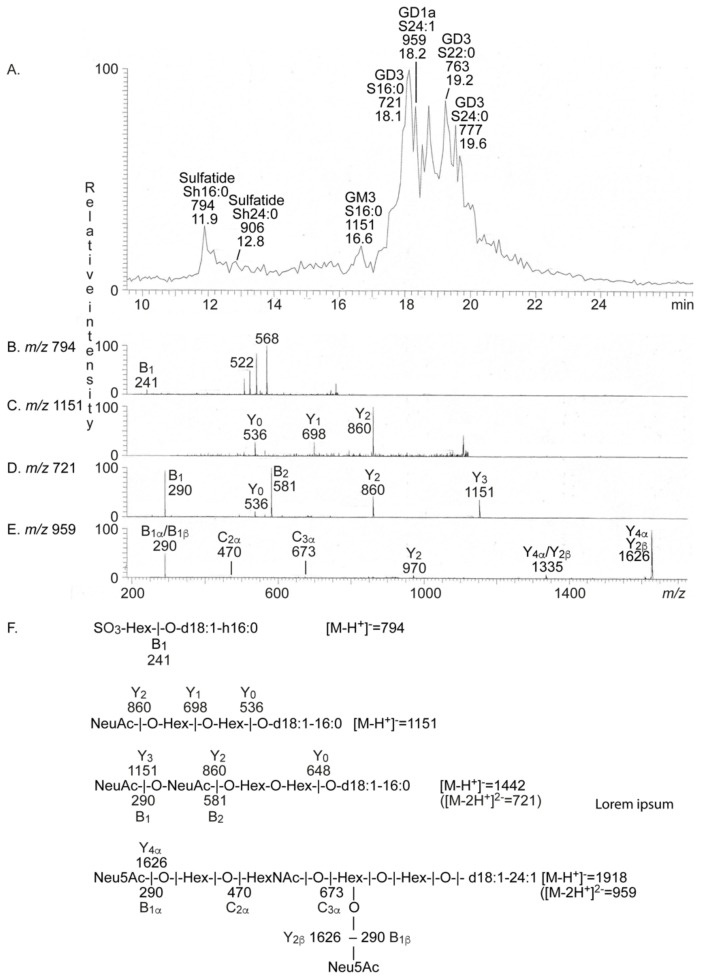
LC-ESI/MS of the total acid glycosphingolipid fraction from human medullary thyroid cancer. The identification of the glycosphingolipids was based on their retention times, determined molecular masses, and subsequent MS^2^ sequencing. (**A**) Base peak chromatogram from LC-ESI/MS of the total acid glycosphingolipid fraction from human medullary thyroid cancer. (**B**) MS^2^ of the ion at *m/z* 794 at retention time 11.9 min. (**C**) MS^2^ of the ion at *m/z* 1151 at retention time 16.5 min. (**D**) MS^2^ of the ion at *m/z* 721 at retention time 18.0 min. (**E**) MS^2^ of the ion at *m/z* 759 at retention time 18.2 min. (**F**) Interpretation formulas. The glycosphingolipids identified in the chromatogram were: Sulfatide, SO_3_-3Galβ1Cer; Neu5Ac-GM3, Neu5Acα3Galβ4Glcβ1Cer; Neu5Ac-GD3, Neu5Acα8Neu5Acα3Galβ4Glcβ1Cer; Neu5Ac-GD1a, Neu5Acα3Galβ3GalNAcβ4(Neu5Acα3)Galβ4Glcβ1Cer. In the shorthand nomenclature for fatty acids and bases, the number before the colon refers to the carbon chain length and the number after the colon gives the total number of double bonds in the molecule. Fatty acids with a 2-hydroxy group are denoted by the prefix h before the abbreviation, as e.g., h16:0. S designates sphingosine (d18:1) long chain base.

The native total acid glycosphingolipid fraction from human medullary thyroid cancer was analyzed by liquid chromatography-electrospray ionization mass spectrometry (LC-ESI/MS). The base peak chromatogram thereby obtained was dominated by doubly charged molecular ions at *m/z* 721, *m/z* 763 and *m/z* 777 (Figure 2A). MS^2^ of these ions (exemplified in Figure 2D) identified the GD3 ganglioside (see Table 1 for acid glycosphingolipid structures), with sphingosine and 16:0, 22:0 and 24:0 fatty acids, respectively.

There were also two singly charged molecular ions at *m/z* 794 and *m/z* 906, and here sulfatide with sphingosine and hydroxy 16:0 and 24:0 fatty acids were characterized by MS^2^ (exemplified in Figure 2B). The GM3 ganglioside with sphingosine and 16:0 fatty acid was characterized by MS^2^ of the singly charged molecular ion at *m/z* 1151 (Figure 2C), and the GD1a ganglioside with sphingosine and 24:1 fatty acid was identified by MS^2^ of the doubly charged molecular ion at *m/z* 959 (Figure 2E). The presence of the GD1a ganglioside was confirmed by the binding of monoclonal anti-GD1a antibodies to the acid glycosphingolipid fraction from human medullary thyroid cancer (Supplementary Figure S1). Binding of monoclonal antibodies directed against sialyl-Le^a^ was also tested but no binding was obtained (data not shown).

The glycosphingolipids characterized in the acid glycosphingolipid fraction are summarized in Table 1.

### 2.3. Characterization of the Non-Acid Glycosphingolipids from Human Medullary Thyroid Cancer

#### 2.3.1. LC-ESI/MS of Glycosphingolipid-Derived Oligosaccharides

The total non-acid glycosphingolipid fraction was hydrolyzed with endoglycoceramidase II from *Rhodococcus* sp., and the oligosaccharides thereby obtained were characterized by LC-ESI/MS using a graphitized carbon column. This method gives a resolution of isomeric oligosaccharides, and by MS^2^ a series of C-type ions is obtained, which gives the carbohydrate sequence [15]. Furthermore, the MS^2^ spectra of oligosaccharides with a Hex or HexNAc substituted at C-4 have diagnostic cross-ring ^0,2^A-type and ^2,4^A-type fragment ions, which allow identification of linkage positions [15,16]. Thus, such fragment ions are present in the MS^2^ spectra of oligosaccharides with globo (Galα4Gal) or type 2 (Galβ4GlcNAc) core structures, but not in the MS^2^ spectra obtained from oligosaccharides with isoglobo (Galα3Gal) or type 1 (Galβ3GlcNAc) core chains. Comparison of retention times and MS^2^ spectra of oligosaccharides from reference glycosphingolipids is also used for identification of oligosaccharides.

**Figure 3:**
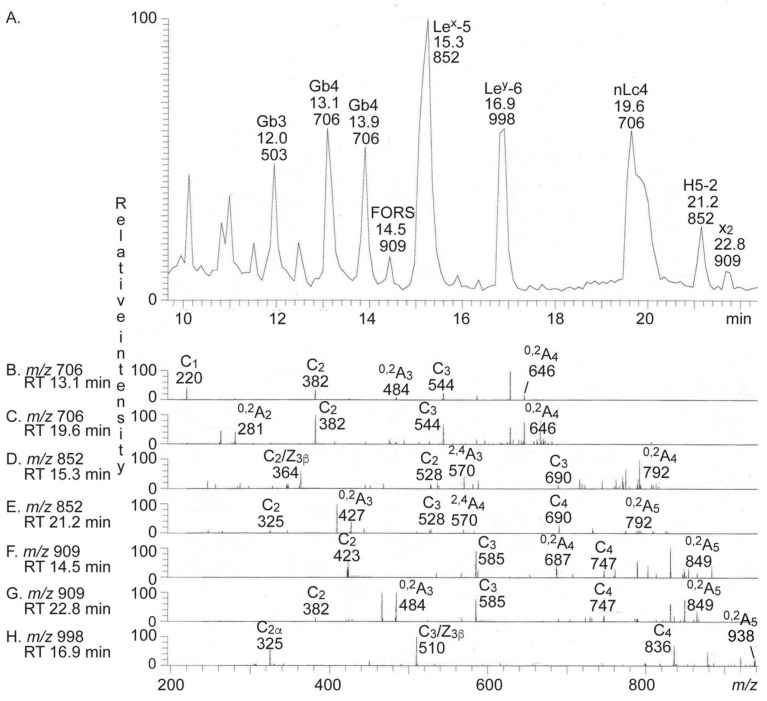
LC-ESI/MS of the oligosaccharides derived from the total non-acid glycosphingolipid fraction from human medullary thyroid cancer by hydrolysis with endoglycoceramidase II from *Rhodococcus* spp. The identification of oligosaccharides was based on their retention times, determined molecular masses, and subsequent MS^2^ sequencing. (**A**) Base peak chromatogram from LC-ESI/MS of the oligosaccharides obtained from the total non-acid glycosphingolipid fraction from human medullary thyroid cancer. (**B**) MS^2^ of the ion at *m/z* 706 at retention time 13.1 min. (**C**) MS^2^ of the ion at *m/z* 706 at retention time 19.6 min. (**D**) MS^2^ of the ion at *m/z* 852 at retention time 15.3 min. (**E**) MS^2^ of the ion at *m/z* 852 at retention time 21.2 min. (**F**) MS^2^ of the ion at *m/z* 909 at retention time 14.5 min. (**G**) MS^2^ of the ion at *m/z* 909 at retention time 22.8 min. (**H**) MS^2^ of the ion at *m/z* 998 at retention time 16.9 min. See Supplementary Figure S2 for interpretation formulas. The oligosaccharides identified in the chromatogram were: Gb3, Galα4Galβ4Glc; Gb4, GalNAcβ3Galα4Galβ4Glc; FORS, GalNAcα3GalNAcβ3Galα4Galβ4Glc; Le^x^-5, Galβ4(Fucα3)GlcNAcβ3Galβ4Glc; Le^y^-6, Fucα2Galβ4(Fucα3)GlcNAcβ3Galβ4Glc; nLc4, Galβ4GlcNAcβ3Galβ4Glc; H5-2, Fucα2Galβ4GlcNAcβ3Galβ4Glc; x_2_, GalNAcβ3Galβ4GlcNAcβ3Galβ4Glc. RT, retention time.

The base peak chromatogram from LC-ESI/MS of the oligosaccharides obtained from the total non-acid glycosphingolipid fraction from human medullary thyroid cancer had a number of molecular ions corresponding to oligosaccharides ranging from trisaccharides (detected as [M − H^+^]^−^ ions at *m/z* 503) to hexasaccharides (detected as [M − H^+^]^−^ ions at *m/z* 998) (Figure 3A). All molecular ions were subjected to MS^2^ and the oligosaccharides thereby identified were given in the chromatogram (see Supplementary Figure S2 for interpretation formulas).

MS^2^ of the ion at *m/z* 503 gave prominent C-type fragment ions (C_1_ at *m/z* 179 and C_2_ at *m/z* 341) identifying a Hex-Hex-Hex sequence (data not shown). A ^2,4^A_2_ fragment ion at *m/z* 221 was present, which demonstrated that the penultimate Hex was 4-substituted. Together this identified a globo trisaccharide (Galα4Galβ4Glc).

The MS^2^ spectrum of the ion at *m/z* 706 at retention time 13.1–13.9 min (Figure 3B) had a C-type fragment ion series (C_1_ at *m/z* 220, C_2_ at *m/z* 382, and C_3_ at *m/z* 544), demonstrating a HexNAc-Hex-Hex-Hex sequence. The ^0,2^A_3_ fragment ion at *m/z* 484 demonstrated a 4-substituted Hex [15,16]. Thereby, a globo tetrasaccharide (GalNAcβ3Galα4Galβ4Glc) was identified.

MS^2^ of the ion at *m/z* 706 at the retention time 19.6 min allowed identification of a neolacto tetrasaccharide (Galβ4GlcNAcβ3Galβ4Glc) (Figure 3C). This was concluded from the C-type fragment ions (C_2_ at *m/z* 382 and C_3_ at *m/z* 544) identifying a Hex-HexNAc-Hex-Hex sequence, along with the prominent ^0,2^A_2_ fragment ion at *m/z* 281 demonstrating a terminal Hex-HexNAc sequence with a 4-substituted HexNAc, i.e., a type 2 chain [15,16].

The major ion in the base peak chromatogram was at *m/z* 852 eluting at 15.3 min. MS^2^ of this ion gave an intense ion at *m/z* 364 (Figure 3D). This fragment ion is obtained by double glycosidic cleavage of the 3-linked branch (C_2_/Z_3β_), and characteristic for an internal 4-linked GlcNAc substituted with a Fuc at 3-position [16]. Together, with the C_2_ ion at *m/z* 528 and the C_3_ ion at *m/z* 690, a Le^x^ pentasaccharide (Galβ4(Fucα3)GlcNAcβ3Galβ4Glc) was thus identified.

The MS^2^ spectrum of the ion at *m/z* 852 eluting at 21.2 min was distinctly different (Figure 3E) and had a series of C type fragment ions (C_2_ at *m/z* 325, C_3_ at *m/z* 528, and C_4_ at *m/z* 690), identifying a pentasaccharide with Fuc-Hex-HexNAc-Hex-Hex sequence. The ^0,2^A_3_ fragment ion at *m/z* 427 is characteristic for 4-substituted HexNAc, i.e., a type 2 carbohydrate chain [15,16]. This demonstrated an H type 2 pentasaccharide (Fucα2Galβ4GlcNAcβ3Galβ4Glc).

The base peak chromatogram had two minor ions at *m/z* 909, eluting at 14.5 min and 22.8 min, respectively. MS^2^ of the ion with retention time 14.5 min (Figure 3F) gave a C type fragment ion series (C_2_ at *m/z* 423, C_3_ at *m/z* 585, and C_4_ at *m/z* 747), identifying a pentasaccharide with HexNAc-HexNAc-Hex-Hex-Hex sequence. In addition, there was a ^0,2^A_4_ fragment ion at *m/z* 687 demonstrating 4-substitution of the internal Hex. This MS^2^ spectrum had a high similarity to the MS^2^ spectrum of the oligosaccharide released from the Forssman pentaosylceramide [17]. Thus, this demonstrated the presence of a Forssman (GalNAcα3GalNAcβ3Galα4Galβ4Glc) or a para-Forssman pentasaccharide (GalNAcβ3GalNAcβ3Galα4Galβ4Glc).

A HexNAc-Hex-HexNAc-Hex-Hex carbohydrate sequence was identified by the series of C type fragment ions (C_2_ at *m/z* 382, C_3_ at *m/z* 585, and C_4_ at *m/z* 747) obtained by MS^2^ of the ion at *m/z* 909 eluting at 22.8 min (Figure 3G). Here, 4-substitution of the internal HexNAc was demonstrated by the ^0,2^A_3_ fragment ion at *m/z* 484. The MS^2^ spectrum was similar to the MS^2^ spectrum of the oligosaccharide released from the x_2_ pentaosylceramide [18] and together demonstrated an x_2_ pentasaccharide (GalNAcβ3Galβ4GlcNAcβ3Galβ4Glc).

Finally, MS^2^ of the ion at *m/z* 998 demonstrated a Le^y^ hexasaccharide (Fucα2Galβ4(Fucα3)GlcNAcβ3Galβ4Glc) (Figure 3H). This conclusion was based on the prominent ion at *m/z* 510, which is obtained by double glycosidic cleavage of the 3-linked branch at C_3_ and Z_3β_, and characteristic for an internal 4-linked GlcNAc substituted with a Fuc at 3-position [16], together with the series of C type fragment ions (C_2α_ at *m/z* 325 and C_4_ at *m/z* 836).

The seven MS^2^ spectra all had ^0,2^A ions, which were derived from cross-ring cleavage of the 4-substituted Glc of the lactose unit at the reducing end.

**Figure 4:**
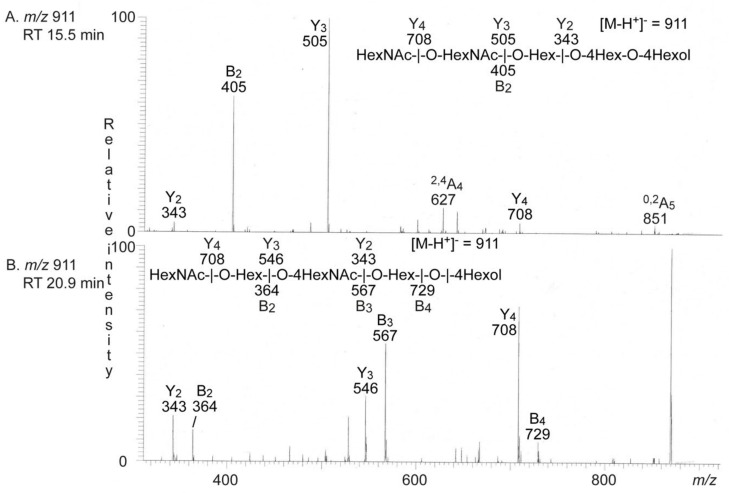
LC-ESI/MS of the reduced oligosaccharides obtained from the total non-acid glycosphingolipid fraction from human medullary thyroid cancer by hydrolysis with endoglycoceramidase II from *Rhodococcus* spp. The identification of the oligosaccharides was based on their retention times, determined molecular masses, and subsequent MS^2^ sequencing. (**A**) MS^2^ of the ion at *m/z* 911 at retention time 15.5 min. (**B**) MS^2^ of the ion at *m/z* 911 at retention time 20.9 min. The interpretation formulas show the deduced oligosaccharide sequences. RT, retention time.

Since the identification of a Forssman (or para-Forssman) pentasaccharide among the oligosaccharides derived from the non-acid glycosphingolipids from human medullary thyroid cancer was an unexpected finding, we next reduced the sample and again, analyzed by LC-ESI/MS. Here two minor ions at *m/z* 911 were present in the base peak chromatogram, eluting at 15.5 min and 20.9 min, respectively. MS^2^ of the ion eluting at 15.5 min (Figure 4A) gave a series of Y ions (Y_2_ at *m/z* 343, Y_3_ at *m/z* 505 and Y_4_ at *m/z* 708) again demonstrating a HexNAc-HexNAc-Hex-Hex-Hex sequence. The HexNAc-HexNAc terminal was further confirmed by the B_2_ at *m/z* 405.

The spectrum obtained by MS^2^ of the ion at *m/z* 911 at 20.9 min (Figure 4B) had a number of Y ions (Y_2_ at *m/z* 343, Y_3_ at *m/z* 546 and Y_4_ at *m/z* 708), and B ions (B_2_ at *m/z* 364, B_3_ at *m/z* 567 and B_4_ at *m/z* 729), in line with a HexNAc-Hex-HexNAc-Hex-Hex sequence.

##### α-*N*-Acetylgalactosaminidase Hydrolysis

Thereafter, the oligosaccharides from non-acid glycosphingolipids fraction were digested with α-*N*-acetylgalactosaminidase to determine if the oligosaccharide with HexNAc-HexNAc-Hex-4Hex-4Hex sequence was derived from a Forssman (GalNAcα3GalNAcβ3Galα4Galβ4Glcβ1Cer) or a para-Forssman (GalNAcβ3GalNAcβ3Galα4Galβ4Glcβ1Cer) glycosphingolipid. The untreated oligosaccharides and the resulting oligosaccharides from the enzymatic digestions were analyzed by LC-ESI/MS (Supplementary Figure S3). Upon treatment with α-*N*-acetylgalactosaminidase the molecular ion at *m/z* 909 disappeared, confirming an α-linked terminal GalNAc as in the Forssman oligosaccharide (Supplementary Figure S3B). The other molecular ions were not affected, except for the molecular ion at *m/z* 1055 (blood group A type 2 hexasaccharide; GalNAcα3(Fucα2)Galβ4GlcNAcβ3Galβ4Glc; see below), which also disappeared upon α-*N*-acetylgalactosaminidase hydrolysis.

#### 2.3.2. Separation of the Non-Acid Glycosphingolipids

**Figure 5:**
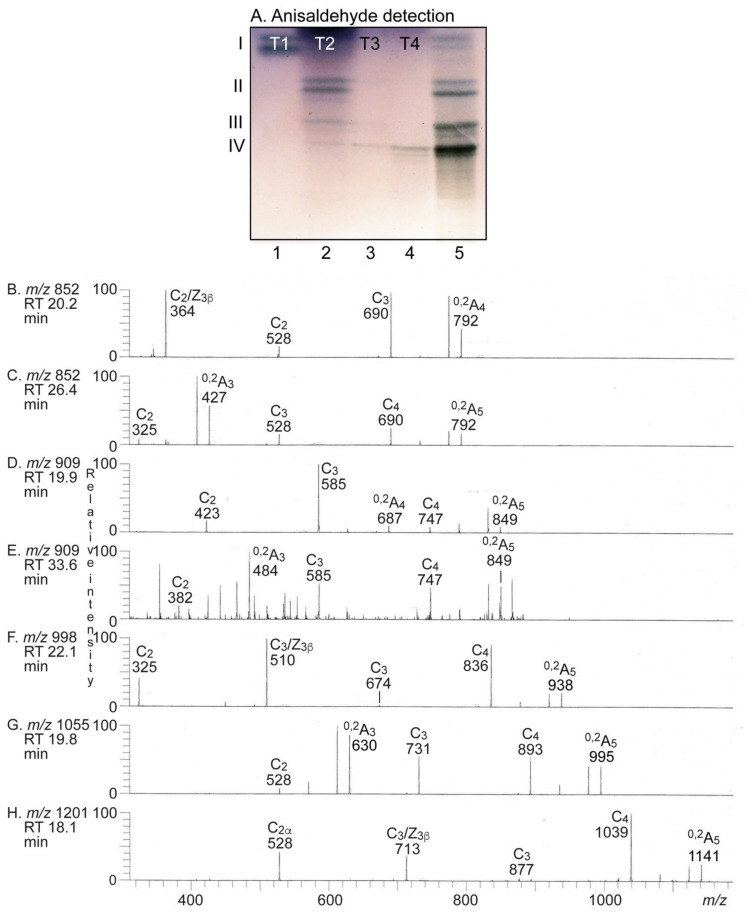
(**A**) Thin-layer chromatography of the non-acid glycosphingolipid subfractions from human medullary thyroid cancer. Thin-layer chromatogram after detection with anisaldehyde. The lanes were: lane 1, glycosphingolipid subfraction T1 from human medullary thyroid cancer, 4 μg; lane 2, subfraction T2, 4 μg; lane 3, subfraction T3, 4 μg; lane 4, subfraction 4, 4 μg; lane 5, reference non-acid glycosphingolipids from human blood group AB erythrocytes, 40 μg. The Roman numbers to the left of the chromatogram indicate the approximate number of carbohydrate units in the bands. (**B**–**H**) LC-ESI/MS of the oligosaccharides obtained from fraction T4 from human medullary thyroid cancer by hydrolysis with endoglycoceramidase II from *Rhodococcus* spp. The identification of the oligosaccharides was based on their retention times, determined molecular masses, and subsequent MS^2^ sequencing. (**B**) MS^2^ of the ion at *m/z* 852 at retention time 20.2 min. (**C**) MS^2^ of the ion at *m/z* 852 at retention time 26.4 min. (**D**) MS^2^ of the ion at *m/z* 909 at retention time 19.0 min. (**E**) MS^2^ of the ion at *m/z* 909 at retention time 33.6 min. (**F**) MS^2^ of the ion at *m/z* 998 at retention time 22.1 min. (**G**) MS^2^ of the ion at *m/z* 1055 at retention time 19.8 min. (**H**) MS^2^ of the ion at *m/z* 1201 at retention time 18.1 min. See Supplementary Figure S2 for interpretation formulas. The oligosaccharides identified were: Le^x^-5, Galβ4(Fucα3)GlcNAcβ3Galβ4Glc; H5-2, Fucα2Galβ4GlcNAcβ3Galβ4Glc; FORS, GalNAcα3GalNAcβ3Galα4Galβ4Glc; x_2_, GalNAcβ3Galβ4GlcNAcβ3Galβ4Glc; Le^y^-6, Fucα2Galβ4(Fucα3)GlcNAcβ3Galβ4Glc; A6-2, GalNAcα3(Fucα2)Galβ4GlcNAcβ3GalβGlc; A7-2, GalNAcα3(Fucα2)Galβ4(Fucα3)GlcNAcβ3Galβ4Glc. RT, retention time.

After these studies, the total non-acid glycosphingolipid fraction from human MTC was separated by chromatography on an latrobeads column, in order to enrich the slow-migrating glycosphingolipids. This gave four subfractions, denoted fractions T1 to T4. The glycosphingolipids in fraction T1 migrated in the monoglycosylceramide region, fraction T2 had compounds migrating as di- to tetraglycosylceramides, fraction T3 mainly tetraglycosylceramides, and fraction T4 had tetraglycosylceramides and more slow-migrating glycosphingolipids (Figure 5A, lanes 1–4)

#### 2.3.3. LC-ESI/MS of Fraction T-1

The native fraction T-1 was analyzed by LC-ESI/MS using a polyamine column (Supplementary Figure S4). Thereby, mono- and dihexosylceramides with sphingosine, and both hydroxy and non-hydroxy fatty acids with 16 and 24 carbon atoms, were identified.

#### 2.3.4. LC-ESI/MS of Fractions T2–T4

Fractions T2, T3 and T4 were hydrolyzed with endoglycoceramidase II and the oligosaccharides obtained were characterized by LC-ESI/MS. The globo trisaccharide, globo tetrasaccharide, and neolacto tetrasaccharide were thereby identified, as above, in fractions T2 and T3 (data not shown). Fraction T3 also had the Le^x^ pentasaccharide and Le^y^ hexasaccharide.

The Le^x^, H type 2, Forssman and x_2_ pentasaccharides, and the Le^y^ hexasaccharide were characterized as described above by LC-ESI/MS of the oligosaccharides obtained from fraction T4 (Figure 5B–F). The base peak chromatogram also had two novel molecular ions at *m/z* 1055 and *m/z* 1201, respectively (Figure 5A). MS^2^ of the ion at *m/z* 1055 gave a series of C-type fragment ions (C_2_ at *m/z* 528, C_3_ at *m/z* 731, and C_4_ at *m/z* 893), which indicated a HexNAc-(Fuc)Hex-HexNAc-Hex-Hex sequence (Figure 5G). The ^0,2^A_3_ fragment ion at *m/z* 630 demonstrated a type 2 core chain. Thus, a blood group A type 2 hexasaccharide (GalNAcα3(Fucα2)Galβ4GlcNAcβ3Galβ4Glc) was identified.

MS^2^ of the molecular ion at *m/z* 1201 (Figure 5H) gave a series of C-type fragment ions (C_2_ at *m/z* 528, C_3_ at *m/z* 877, and C_4_ at *m/z* 1039), which demonstrated a HexNAc-(Fuc-)Hex-(Fuc-)HexNAc-Hex-Hex sequence. The spectrum also had a fragment ion at *m/z* 713, which is obtained by double glycosidic cleavage of the 3-linked branch (C_3_/Z_3β_), and thus characteristic for an internal 4-linked GlcNAc substituted with a Fuc at 3-position [19]. Together, this identified a blood group A type 2/ALe^y^ heptasaccharide (GalNAcα3(Fucα2)Galβ4(Fucα3)GlcNAcβ3Galβ4Glc).

**Figure 6:**
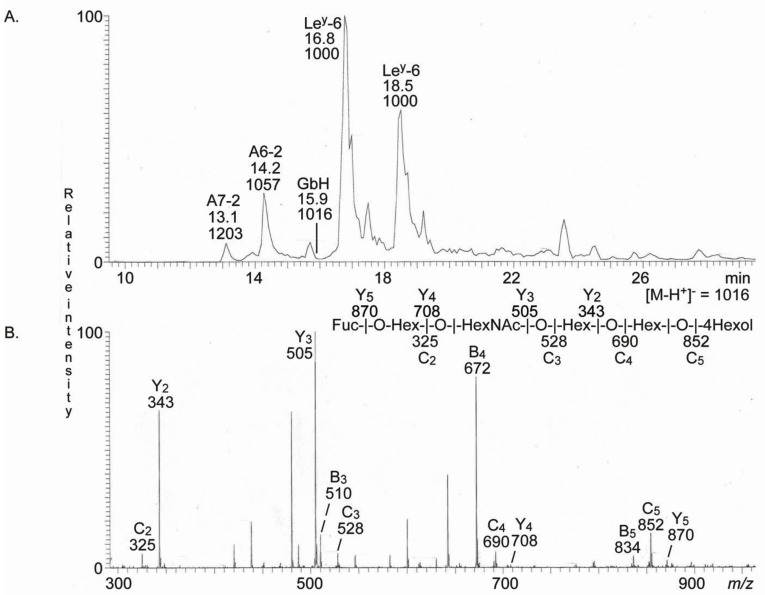
LC-ESI/MS (*m/z* 1000–2000) of the reduced oligosaccharides obtained from fraction T2-4 from human medullary thyroid cancer by hydrolysis with endoglycoceramidase II from *Rhodococcus* spp. The identification of the oligosaccharides was based on their retention times, determined molecular masses, and subsequent MS^2^ sequencing. (**A**) Base peak chromatogram from LC-ESI/MS of the reduced oligosaccharides obtained from fraction T4. (**B**) MS^2^ of the ion at *m/z* 1016 at retention time 15.9 min. The interpretation formula shows the deduced oligosaccharide sequence. The oligosaccharides identified in the chromatogram were: A7-2, GalNAcα3(Fucα2)Galβ4(Fucα3)GlcNAcβ3Galβ4Glc; A6-2, GalNAcα3(Fucα2)Galβ4GlcNAcβ3GalβGlc; GbH, Fucα2Galβ3GalNAcβ3Galα4Galβ4Glc; Le^y^-6, Fucα2Galβ4(Fucα3)GlcNAcβ3Galβ4Glc.

In a final attempt to identify minor complex compounds, we re-analyzed the oligosaccharides at *m/z* 1000–2000. Therefore, a minor molecular ion at *m/z* 1016 (reduced *m/z* 1014) was detected when analyzing the reduced oligosaccharides from fractions T4 (Figure 6A). MS^2^ of this ion (Figure 6B) gave series with B and C type fragment ions (C_2_ at *m/z* 325, B_3_ at *m/z* 510, C_3_ at *m/z* 528, B_4_ at *m/z* 672, C_4_ at *m/z* 690, B_5_ at *m/z* 834, and C_5_ at *m/z* 852) and a Y type ion series (Y_2_ at *m/z* 343, Y_3_ at *m/z* 505, Y_4_ at *m/z* 708, and Y_5_ at *m/z* 870), establishing a Fuc-Hex-HexNAc-Hex-Hex-Hex carbohydrate sequence. Thus, the Globo H oligosaccharide (Fucα2Galβ3GalNAcβ3Galα4Galβ4Glc) was tentatively identified.

#### 2.3.5. Binding of Carbohydrate Recognizing Ligands to the Non-Acid Glycosphingolipids

**Figure 7:**
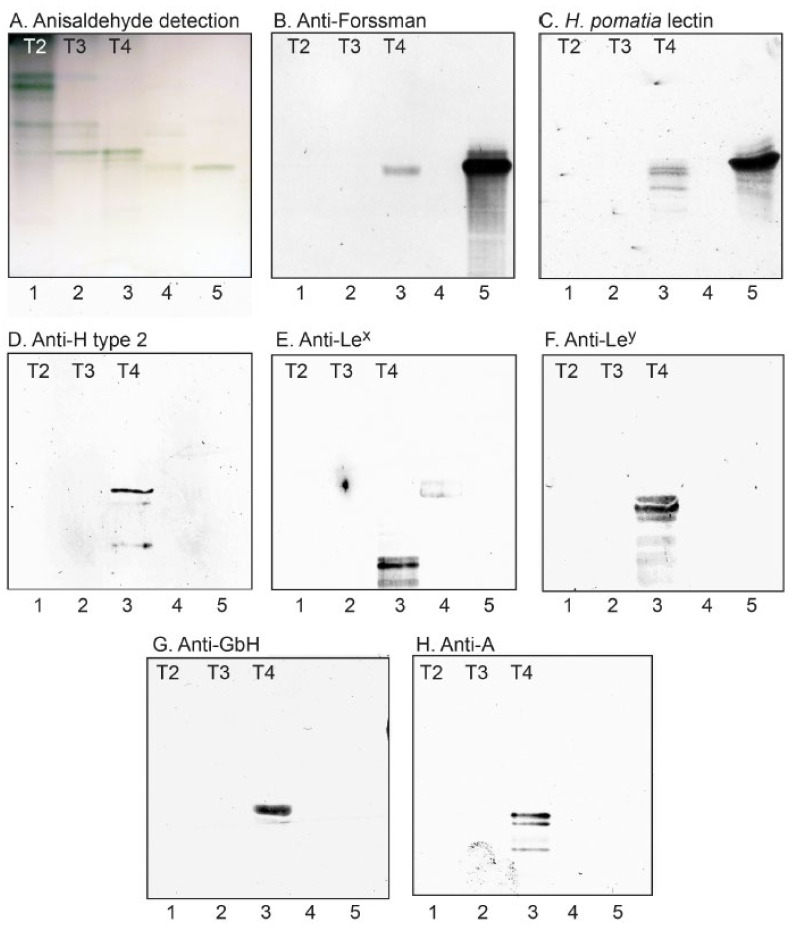
Binding of lectins and antibodies to the non-acid glycosphingolipid subfractions from human medullary thyroid cancer. Thin-layer chromatogram after detection with anisaldehyde (**A**), and autoradiograms obtained by binding of monoclonal anti-Forssman antibodies (**B**), GalNAcα-binding *H. pomatia* lectin (**C**), monoclonal anti-H type 2 antibodies (**D**), monoclonal anti-Le^x^ antibodies (**E**), monoclonal anti-Le^y^ antibodies (**F**), monoclonal anti-globo H antibodies (**G**), and monoclonal anti-A antibodies (**H**). The lanes were: lane 1, glycosphingolipid subfraction T2 from human medullary thyroid cancer, 4 μg; lane 2, subfraction T3, 4 μg; lane 3, subfraction T4, 4 μg; lane 4, reference neolactotetraosylceramide (Galβ4GlcNAcβ3Galβ4Glcβ1Cer), 2 μg, and reference Le^x^ pentaosylceramide (Galβ4(Fucα3)GlcNAcβ3Galβ4Glcβ1Cer), 2 μg; lane 5, reference Forssman pentaosylceramide (GalNAcα3GalNAcβ3Galα4Galβ4Glcβ1Cer), 4 μg.

In order to validate the structural information obtained by mass spectrometry, the binding of carbohydrate binding ligands to fractions T2–T4 was next examined in a chromatogram binding assay (Figure 7). Thereby, a distinct interaction of the GalNAcα binding lectin from *H. pomatia* [20], and the anti-Forssman monoclonal antibodies, to fraction T4 was obtained (Figure 7B,C, lane 3), confirming the presence of the Forssman glycosphingolipid. The existence of the H type 2 pentaosylceramide in fraction T4 was confirmed by the binding of anti-H type 2 monoclonal antibodies (Figure 7D, lane 3), and these antibodies also marked the H type 2 heptaosylceramide. The presence of glycosphingolipids with blood group Le^x^, Le^y^, Globo H and A determinants in fraction T4 was confirmed by the binding of monoclonal antibodies (Figure 7E–H, lane 3). The antibodies against blood group Le^x^, Le^y^, and A determinants also recognized slow-migrating compounds in fraction T4, indicating the presence of complex glycosphingolipids carrying these determinants.

The oligosaccharides derived from the non-acid glycosphingolipids of human medullary thyroid cancer are summarized in Table 2.

### 2.4. Immunohistochemistry

**Figure 8:**
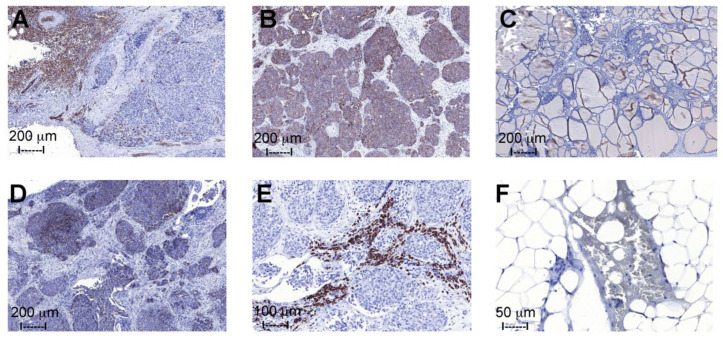
Immunohistochemical evaluation of medullary thyroid cancer (**A**,**B**,**D**–**F**) and non-neoplastic follicular thyroid tissue samples (**C**). (**A**) shows a positive staining with the anti-A antibody in the supportive tissue and in some of the tumor cells, (**B**) shows an extensive expression of Le^y^ antigens in the medullary thyroid cancer cells, (**C**) shows no significant staining of benign/non-neoplastic thyroid tissue with the anti-Le^y^ antibody, (**D**) shows the expression of the Globo H antigen in a subpopulation of the tumor cells, (**E**) shows a positive staining with the anti-Le^x^ antibody in a portion of the tumor cells, and (**F**) shows the expression of Forssman antigens in the tumor and blood cells of one individual with blood group B.

Immunohistochemical analysis demonstrated expression of blood group A antigens in the MTC samples collected from patients with blood group A (*n* = 2) (Figure 8A), and no presence in the blood group B (*n* = 1) and O (*n* = 3) individuals. An evident blood group A expression was seen in the vascular and supportive tissue, and in some of the tumor cells. All MTC cells extensively expressed the Le^y^ determinant (Figure 8B), while no significant positive staining was seen in the supportive tissue or in the non-neoplastic follicular thyroid tissue (Figure 8C). The extent and intensity of the noted positive staining with anti-Globo H (Figure 8D) and anti-Le^x^ (Figure 8E) antibodies was variable, but clearly present, in all samples demonstrating a subpopulation of cells with antigen expression. One individual expressed the Forssman antigens in the tumor and blood cells (Figure 8F), all other samples (*n* = 8) showed no positive staining with the anti-Forssman antibody.

## 3. Discussion

In this study, we have obtained a high resolution of the glycosphingolipids present in human medullary carcinomas of the thyroid. This was due to a large amount of starting material (wet weight 20 g/dry weight 5.4 g) allowing several chromatographic purification steps, and separation of the glycosphingolipids into total acid and non-acid fractions. The glycosphingolipid material obtained also permitted us to obtain partly purified glycosphingolipid subfractions, which made it possible to identify minor compounds using a combination of mass spectrometry and binding of carbohydrate recognizing ligands.

The GD3 ganglioside was the acid major glycosphingolipid, as previously reported by Mariano et al. [12]. The acid fraction also had sulfatide, and the gangliosides GM3 and GD1a (see Table 1 for glycosphingolipid structures). The amount of acid glycosphingolipids in the medullary thyroid cancers was higher than in the normal thyroid glands. This is in agreement with previous lectin immunohistochemistry studies, which showed an increased sialylation in thyroid cancers, albeit mainly in papillary and follicular carcinomas [21]. However, other studies have reported that malignant transformation in the thyroid gland leads to decreased sialylation [22,23].

A weak expression of the sialyl-Le^a^ antigen (CA19:9 ligand) in a few MTC has been reported [22,23]. In our study, we did not find sialyl-Le^a^ by mass spectrometry, and no binding of the 19:9 antibody to the acid glycosphingolipids was obtained.

For the characterization of the non-acid glycosphingolipids, we used endoglycoceramidase II from *Rhodococcus* sp., and the oligosaccharides thereby released were analyzed by LC/MS. Here, it should be noted that the relative intensities of the molecular ions in the chromatograms do not mirror the relative abundance of the glycosphingolipids in the samples, since the hydrolytic capacity of endoglycoceramidase II is somewhat restricted, certain glycosphingolipods, e.g., globo-series glycosphingolipids, are relatively resistant to this enzyme [24,25].

Among the non-acid glycosphingolipids, the majority of the characterized complex compounds had type 2 (Galβ4GlcNAc) core chains, i.e., the neolactotetraosylceramide, the Le^x^, H type 2 and x_2_ pentaosylceramides, the Le^y^ and A type 2 hexaosylceramides, and the A type 2 heptaosylceramide. In addition, there were compounds with globo (GalαGalβ4Glc) core, i.e., globotriaosylceramide, globotetraosylceramide, the Forssman pentaosylceramide, and the globo H hexaosylceramide (see Table 2 for oligosaccharide structures). No compounds with type 1 (Galβ3GlcNAc) or ganglio (Galβ3GalNAc) core chains were characterized.

These findings are supported by the outcome of the immunohistochemistry analysis, which showed expression of blood group A and Le^x^ determinants in a portion of the MTC cells, and extensive expression of the Le^y^ antigens in all MTC samples. However, only five patients were studied here, so further immunohistochemistry analysis should be carried out. The Le^y^ antigen is upregulated on many types of cancer, e.g., lung, breast, colorectal, ovarian, and prostate cancers, and is a promising target for gene-modified T cells and antibody targeting reviewed in [8]. Thus, Le^y^ may potentially be used in the case of MTC as a target for the development of future therapeutic and diagnostic applications.

Globo H is a prevalent cancer-associated glycosphingolipid and is overexpressed in many cancers of epithelial origins (e.g., breast, ovary, uterus, prostate, lung, colon, and liver cancers) [26]. Currently, there are several ongoing clinical trials on immunotherapies targeting globo H. Immunohistochemistry studies have demonstrated the presence of Globo H in 33% of MTCs, but not in normal thyroidea or benign thyroid lesions [27]. In our study, the Globo H hexaosylceramide was minor, and by mass spectrometry, it could only be characterized by a targeted search. Immunohistochemical evaluation of nine MTC samples showed a subpopulation, of variable quantity, that clearly expressed Globo H in all of the examined individuals. Hence, it would be of great interest to investigate the outcome of immunotherapy against Globo H in MTC patients.

The characterization of the Forssman pentaosylceramide among the non-acid glycosphingolipids from human medullary thyroid cancer was an unexpected finding. Further investigation with immunohistochemistry of samples from five of the 14 individuals included in the glycosphingolipid preparation identified one individual with blood group B, and diagnosed an individual with multiple endocrine neoplasia type 2B (MEN2B) expressing Forssman antigens. However, the sample from this individual was a small lymph node with a high content of adipocytes and a limited amount of tumor tissue.

Until recently, humans were considered to be a Forssman negative species since the Forssman synthase gene in humans is inactive. However, the identification of the Forssman glycolipid antigen on erythrocytes of the rare individuals of the A_pae_ phenotype led to the recognition of the FORS system as a novel blood group system [17]. In these A_pae_ individuals, the gene coding for the Forssman synthase (*GBGT1*) encodes an arginine to glutamine change at residue 296, which gives reactivation of the human Forssman synthase. FORS is, however, a rare blood group system, and some individuals have naturally occurring anti-Forssman (anti-FORS1) antibodies in plasma. However, the frequency of these anti-FORS1 antibodies is still unclear [28,29].

A role for Forssman as a tumor-associated carbohydrate antigen has not yet been established. During the 1970ies–1980ies the presence of the Forssman glycosphingolipid both in normal human tissues as kidney and gastrointestinal mucosa [30,31], and in some human cancers as e.g., gastric, lung and liver cancer, was reported (reviewed in [32]). However, if this was the product of the Forssman synthase is not known. Furthermore, some of these studies used polyclonal sera for detection of Forssman, and thus cross-reactions with other glycans are possible.

More recently it was demonstrated that vaccination with the terminal disaccharide of the Forssman antigen (GalNAcα3GalNAcβ) induce antibody responses to the Forssman disaccharide, and those responses correlate with long-term survival of vaccinated patients with prostate cancer [33].

The finding of Forssman expression in a patient with MEN2B is an intriguing combination since both are very rare events in the clinic. We are now investigating the molecular background, and the potential association, which will be reported separately.

The expression of blood group antigens in MTC has been investigated by immunohistochemistry studies using monoclonal antibodies [34,35,36]. These studies demonstrated that blood group antigens were not expressed in normal follicular or C-cells, but were expressed to a various extent in medullary thyroid cancers, and were expressed more frequently in malignant than in benign neoplasms. The occurrence blood group antigens with type 1 core chain in medullary carcinomas of the thyroid has been demonstrated [34]. These findings should reflect binding to glycoproteins since no type 1 core glycosphingolipids were characterized in our current study. However, it should be kept in mind that monoclonal antibodies against carbohydrate antigens often are highly cross-reactive [37], and may give false-positive results leading to an overestimation of antigen expression levels. Thus, verification of the results with one or more independent analytical methods is needed.

No tumor specific biomarkers are currently available for diagnostics, prognostic evaluation, or treatment monitoring of medullary thyroid cancer. In this study of the glycosphingolipids of MTC the most intriguing findings were the presence of Le^y^, Le^x^, globo H and the Forssman glycosphingolipids. These aberrations in the glycan structures suggest the potential utility of these compounds as markers for medullary thyroid cancer.

## 4. Materials and Methods

### 4.1. Glycosphingolipid Preparations

This study was performed on MTCs from patients treated at the Department of Surgery, Uppsala University Hospital, Sweden and included biomaterial from Uppsala Biobank, Endocrine tumor collection (Ethical approval 00-128/3.15.2000). Clinical data were extracted through patient chart reviews. The study was approved by the Swedish Ethical Review Authority (No. 2020-06142) and followed the Declaration of Helsinki and the General Data Protection Regulation (GDPR). All patients were given written and verbal information before signing informed consent to participate and agreeing to the use of the information in research.

For the glycosphingolipid analysis, 20 g of pooled tumor material from 14 different patients was used. The tissues were obtained at surgery. The ABO blood group status of the patients (A (*n* = 2), O (*n* = 2) and B (*n* = 1)) was routinely established prior to surgery.

The pooled MTC tissue was kept at −70 °C. The pooled material was lyophilized, giving dry weight 5.4 g. Isolation of total acid and total non-acid glycosphingolipids was conducted by the method described by Karlsson [13]. The lyophilized material was extracted in two steps in a Soxhlet apparatus with chloroform and methanol (2:1 and 1:9, by volume, respectively). The extract was subjected to mild alkaline hydrolysis and dialysis, followed by separation on a silicic acid column. Acid and non-acid glycosphingolipid fractions were obtained by chromatography on a DEAE-cellulose column. In order to separate the non-acid glycosphingolipids from alkali-stable phospholipids, the non-acid fractions were acetylated and separated on a second silicic acid column, followed by deacetylation and dialysis. Final purifications were carried out by chromatographies on DEAE-cellulose and silicic acid columns.

The amounts of total acid and non-acid glycosphingolipids obtained are provided in Supplementary Table S1.

The total glycosphingolipid fractions were characterized by liquid chromatography-electrospray mass spectrometry (LC-ESI/MS), thin-layer chromatography, and binding of carbohydrate recognizing ligands (antibodies, lectins, and bacteria) in chromatogram binding assays (see below).

Thereafter, the total non-acid glycosphingolipid fraction was separated by chromatography on an Iatrobeads (Iatron Labs. Inc., Tokyo, Japan; Iatrobeads 6RS-8060) column eluted with increasing amounts of methanol in chloroform. This gave four subfractions, which were denoted fractions T1 to T4.

### 4.2. Reference Glycosphingolipids

Total acid and non-acid glycosphingolipid fractions were isolated as described [13]. Individual glycosphingolipids were isolated by repeated chromatography on silicic acid columns and by HPLC, and identified by mass spectrometry [15,38] and ^1^H-NMR spectroscopy [39].

### 4.3. Thin-Layer Chromatography

Thin-layer chromatography was carried out on aluminum- or glass-backed silica gel 60 high performance thin-layer chromatography plates (Merck, Darmstadt, Germany; 105641/105547). Glycosphingolipid mixtures (40 μg) or pure glycosphingolipids (2–8 μg) were applied to the plates, and eluted with chloroform/methanol/water 60:35:8 (by volume). Chemical detection was carried out with anisaldehyde [40].

### 4.4. Chromatogram Binding Assays

The mouse monoclonal antibodies tested for binding to the glycosphingolipids of human MTC in the chromatogram binding assay are provided in Supplementary Table S2. Binding of the monoclonal antibodies to glycosphingolipids separated on thin-layer chromatograms was carried out as described [41,42]. Chromatograms with separated glycosphingolipids were dipped for 1 min in diethylether/*n*-hexane (1:5, by volume) containing 0.5% (*w*/*v*) polyisobutylmethacrylate (Sigma-Aldrich, St. Louis, MO, USA; 100185005B). After drying, the chromatograms were soaked in PBS containing 2% bovine serum albumin and 0.1% NaN_3_ (Solution A), for 2 h at room temperature. Suspensions of primary monoclonal antibodies diluted 1:100–1:500 (the dilution used for each monoclonal antibody is given in Supplementary Table S2) in Sol. A were gently sprinkled over the chromatograms, followed by incubation for 2 h at room temperature, followed by washings with PBS. For detection two types of secondary antibodies were used. Firstly, ^125^I-labeled (labeled by the Iodogen method according to the manufacturer’s (Pierce/Thermo Fischer Scientific, Stockholm, Sweden; 28600) instructions) rabbit anti-mouse antibodies were diluted to 2 × 10^6^ cpm/mL in Solution A, which were incubated for 2 h. Thereafter, the plates were washed six times with PBS, Dried chromatograms were then autoradiographed for 12–24 h using XAR-5 X-ray films (Carestream/Sigma-Aldrich, St. Louis, MO, USA; 8941114). Other types of secondary antibodies used were alkaline phosphate-conjugated goat anti-mouse antibodies (Sigma-Aldrich, St. Louis, MO, USA; A0162) at a dilution of 1:500, which were incubated for 1 h. Thereafter the reactions were visualized with 5-bromo-4-chloro-3-indolyl phosphate/nitro blue tetrazolium chromogenic substrate (Sigma-Aldrich, St. Louis, MO, USA; B5655-25TAB). Binding of ^125^I-labeled *Helix pomatia* lectin (Sigma-Aldrich, St. Louis, MO, USA; L3382) to glycosphingolipids on thin-layer chromatograms was carried out as described [43].

### 4.5. LC-ESI/MS of Native Acid Glycosphingolipids

The native acid glycosphingolipid fractions were analyzed by LC-ESI/MS as described [44]. Aliquots of the glycosphingolipid fractions were dissolved in methanol:acetonitrile in proportion 75:25 (by volume) and separated on a 200 × 0.250 mm column, packed in-house with 5 μm polyamine II particles (YMC Europe GmbH, Dinslaken, Germany). An autosampler, HTC-PAL (CTC Analytics AG, Zwingen, Switzerland) equipped with a cheminert valve (0.25 mm bore) and a 2 µL loop, was used for sample injection. An Agilent 1100 binary pump (Agilent technologies, Palo Alto, CA, USA) delivered a flow of 250 µL/min, which was split in an 1/16” microvolume-T (0.15 mm bore) (Vici AG International, Schenkon, Switzerland) by a 50 cm × 50 µm i.d. fused silica capillary before the injector of the autosampler, allowing approximately 2–3 µL/min through the column. Samples were eluted with an aqueous gradient (A:100% acetonitrile to B: 10 mM ammonium bicarbonate). The gradient (0–50% B) was eluted for 40 min, followed by a wash step with 100% B, and equilibration of the column for 20 min. The samples were analyzed in negative ion mode on a LTQ linear quadropole ion trap mass spectrometer (Thermo Electron, San José, CA, USA), with an IonMax standard ESI source equipped with a stainless steel needle kept at −3.5 kV. Compressed air was used as nebulizer gas. The heated capillary was kept at 270 °C, and the capillary voltage was −50 kV. Full scan (*m/z* 500–1800, two microscan, maximum 100 ms, target value of 30,000) was performed, followed by data-dependent MS^2^ scans (two microscans, maximun 100 ms, target value of 10.000) with normalized collision energy of 35%, isolation window of 2.5 units, activation q = 0.25 and activation time 30 ms). The threshold for MS^2^ was set to 500 counts.

Data acquisition and processing were conducted with Xcalibur software (Version 2.0.7). Manual assignment of glycosphingolipid sequences was carried out with the assistance of the Glycoworkbench tool (Version 2.1), and by comparison of retention times and MS^2^ spectra of reference glycosphingolipids.

### 4.6. Endoglycoceramidase Digestion and LC-ESI/MS

Endoglycoceramidase II from *Rhodococcus* spp. (Takara Bio Europe S.A., Gennevilliers, France) was used for hydrolysis of the non-acid glycosphingolipids. The glycosphingolipids (50 μg) were resuspended in 100 μL 0.05 M sodium acetate buffer, pH 5.0, containing 120 μg sodium cholate, and sonicated briefly. Thereafter, 1 mU of enzyme was added, and the mixture was incubated at 37 °C for 48 h. The reaction was stopped by addition of chloroform/methanol/water to the final proportions 8:4:3 (by volume). The oligosaccharide-containing upper phase thus obtained was separated from detergent on a Sep-Pak QMA cartridge (Waters). The eluant containing the oligosaccharides was dried under nitrogen and under vacuum.

Part of the oligosaccharide samples were reduced by adding 20 μL of 200 mM NaBH_4_ in 50 mM KOH to the samples and incubating at 50 °C for 2 h [15]. The samples were then acidified by adding 10 μL of glacial acetic acid, and the oligosaccharides were desalted by cation exchange chromatography, and thereafter evaporated to dryness.

The glycosphingolipid-derived oligosaccharides were resuspended in 50 μL water and analyzed by LC-ESI/MS as described [15]. The oligosaccharides were separated on a column (100 × 0.250 mm) packed in-house with 5 μm porous graphite particles (Hypercarb, Thermo-Hypersil, Runcorn, UK). An autosampler, HTC-PAL (CTC Analytics AG, Zwingen, Switzerland) equipped with a cheminert valve (0.25 mm bore) and a 2 μL loop, was used for sample injection. An Agilent 1100 binary pump (Agilent technologies, Palo Alto, CA, USA) delivered a flow of 250 μL/min, which was split down in an 1/16” microvolume-T (0.15 mm bore) (Vici AG International, Schenkon, Switzerland) by a 50 cm × 50 μm i.d. fused silica capillary before the injector of the autosampler, allowing approximately 3–5 μL/min through the column. The oligosaccharides (3 μL) were injected on to the column and eluted with an acetonitrile gradient (A: 10 mM ammonium bicarbonate; B: 10 mM ammonium bicarbonate in 80% acetonitrile). The gradient (0–45% B) was eluted for 46 min, followed by a wash step with 100% B, and equilibration of the column for 24 min. A 30 cm × 50 μm i.d. fused silica capillary was used as transfer line to the ion source.

The oligosaccharides were analyzed in negative ion mode on an LTQ linear quadrupole ion trap mass spectrometer (Thermo Electron, San José, CA, USA). The IonMax standard ESI source on the LTQ mass spectrometer was equipped with a stainless steel needle kept at −3.5 kV. Compressed air was used as nebulizer gas. The heated capillary was kept at 270 °C, and the capillary voltage was −50 kV. Full-scan (*m/z* 380–2000, 2 microscans, maximum 100 ms, target value of 30,000) were performed, followed by data dependent MS^2^ scans of the three most abundant ions in each scan (2 microscans, maximum 100 ms, target value of 10,000). The threshold for MS^2^ was set to 500 counts. Normalized collision energy was 35%, and an isolation window of 3 u, an activation *q* = 0.25, and an activation time of 30 ms, was used. Data acquisition and processing were conducted with Xcalibur software (Thermo Scientific, Waltham, MA, USA; Version 2.0.7)).

Manual assignment of glycan sequences was carried out on the basis of knowledge of mammalian biosynthetic pathways, with the assistance of the Glycoworkbench tool (Version 2.1), [45] and by comparison of retention times and MS^2^ spectra of oligosaccharides from reference glycosphingolipids [15]. The raw data files were deposited in Glycopost: https://glycopost.glycosmos.org/entry/GPST000197, accessed on 3 June 2021.

#### α-*N*-Acetylgalactosaminidase Hydrolysis

The oligosaccharides from total non-acid glycosphingolipid fraction were dissolved in 20 μL 100 mM sodium citrate phosphate buffer (pH 5.0) and digested with 1.5 mU chicken liver α-*N*-acetylgalactosaminidase (Prozyme/Agilent Technologies Sweden AB, Kista, Sweden; GKX5001) at 37 °C overnight. The sample was cleaned up with Hypersep Hypercarb (Thermo Scientific, Waltham, MA, USA; 60106-301), according to the manufacturer’s instructions. Thereafter, the oligosaccharides were analyzed by LC-ESI/MS.

### 4.7. Histology and Immunohistochemistry

For immunohistochemistry, paraffin embedded sections from nine tumor samples (five lymph node metastases and four primary MTC tissues) from five different patients with MTC were utilized (see Supplementary Table S3 for patient characteristics).

Nine MTC samples from the five different patients were included in the pooled material for glycosphingolipid preparation, and three non-neoplastic follicular thyroid tissue samples, were fixed in buffered 4% paraformaldehyde, dehydrated, and embedded in paraffin. Subsequently, 4 µm sections were mounted on Superfrost Plus glass slides (VWR, Radnor, PA, USA; 630-0951) and microwave treated for antigen retrieval. Immunostaining was performed after pretreatment with Diva Decloaker 20X (Biocare Medical, Pacheco, CA, USA; DV2005 L2J) at 95 °C for 40 min. The primary antibodies used (see Supplementary Table S2) were anti-Forssman, anti-blood group A, anti-Globo H, anti-Le^x^, and anti-Le^y^, all diluted 1:50. Rat HRP-polymer kit (Biocare Medical RT517) or MACH4 Universal HRP-polymer detection system (Biocare Medical; M4U534, together with betazoid DAB substrate kit (Biocare Medical; BDB2004) was used for detection of bound antibodies. Nuclei were counterstained with Tacha’s automated hematoxylin (Biocare Medical; NM-HEM).

## Figures and Tables

**Table 1:** Glycosphingolipids identified by LC-ESI/MS in the total acid fraction from human medullary thyroid cancer.

*m/z*	Trivial Name	Structure
794	Sulfatide	SO_3_-3Galβ1Cer
1151	Neu5Ac-GM3	Neu5Acα3Galβ4Glcβ1Cer
721	Neu5Ac-GD3	Neu5Acα8Neu5Acα3Galβ4Glcβ1Cer
959	Neu5Ac-GD1a	Neu5Acα3Galβ3GalNAcβ4(Neu5Acα3)Galβ4Glcβ1Cer

**Table 2:** Glycosphingolipid-derived oligosaccharides from the non-acid fractions from human medullary thyroid cancer identified by LC-ESI/MS.

*m/z*	Trivial Name	Structure
503	Globotri (Gb3)	Galα4Galβ4Glc
706	Neolactotetra (nLc4)	Galβ4GlcNAcβ3Galβ4Glc
706	Globotetra (Gb4)	GalNAcβ3Galα4Galβ4Glc
909	Forssman	GalNAcα3GalNAcβ3Galα4Galβ4Glc
909	x_2_ penta (x_2_)	GalNAcβ3Galβ4GlcNAcβ3Galβ4Glc
852	H type 2 penta (H5-2)	Fucα2Galβ4GlcNAcβ3Galβ4Glc
852	Le^x^ penta (Le^x^-5)	Galβ4(Fucα3)GlcNAcβ3Galβ4Glc
998	Le^y^ hexa (Le^y^-6)	Fucα2Galβ4(Fucα3)GlcNAcβ3Galβ4Glc
1016 (red) *	Globo H	Fucα2Galβ3GalNAcβ3Galα4Galβ4Glc
1055	A hexa type 2 (A6-2)	GalNAcα3(Fucα2)Galβ4GlcNAcβ3Galβ4Glc
1201	A hepta type 2 (A7-2)	GalNAcα3(Fucα2)Galβ4(Fucα3)GlcNAcβ3Galβ4Glc

* Reduced.

## Data Availability

The raw data files from LC-ESI/MS were deposited in Glycopost: https://glycopost.glycosmos.org/entry/GPST000197, accessed on 3 June 2021.

## Supplementary Materials

The following are available online at https://www.mdpi.com/article/10.3390/ijms221910463/s1.

## Abbreviations

MEN2B: multiple endocrine neoplasia type 2B; MTC, medullary thyroid carcinoma; LC-ESI/MS, liquid chromatography electrospray ionization mass spectrometry.
