# Supplementary material for: Characterization of Human Medullary Thyroid Carcinoma Glycosphingolipids Identifies Potential Cancer Markers
Source: Int J Mol Sci. 2021 Sep 28;22(19):10463. doi: 10.3390/ijms221910463 (PMC8509059; doi:10.3390/ijms221910463)
Supplement: Supplementary file 1 [file ijms-22-10463-s001.zip › ijms-1392159-supplementary.pdf]

SUPPLEMENTARY MATERIAL

Characterization of Human Medullary Thyroid Carcinoma Glycosphingolipids Identifies Potential Cancer Markers

Karin Säljö, Anders Thornell, Chunsheng Jin, Olov Norlén, and Susann Teneberg

Supplementary figures

Fig. S1

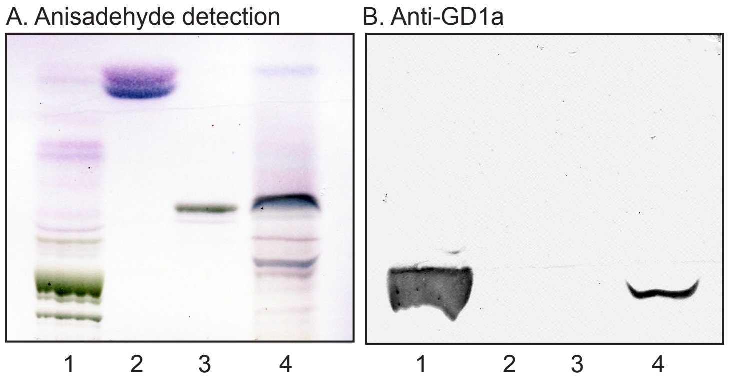

Supplementary Figure S1. Binding of monoclonal anti-GD1a antibodies to the acid glycosphingolipids from human medullary thyroid cancer. Thin-layer chromatogram after detection with anisaldehyde (A), and autoradiogram obtained by binding of monoclonal anti-GD1a antibodies (B). The lanes were: lane 1, calf brain gangliosides , 40 µg; lane 2, sulfatide (SO_3-3Galβ1Cer), 4 µg; lane 3, GM3 ganglioside (Neu5Acα3Galβ4Glcβ1Cer), 4 µg; acid glycosphingolipids from human medullary thyroid cancer, 40 µg.

Fig. S2

```
A. m/z 706    HexNAc-O-|-Hex-O-|-4Hex-O-|-4Hex                    Globotetra
                       220      382      544
                       C1       C2       C3

B. m/z 706    Hex-O-4HexNAc-O-|-Hex-O-|-4Hex                      Neolactotetra
                              382     544
                              C2      C3

C. m/z 852    Hex-O-4HexNAc-O-|-Hex-O-|-4Hex                      Lex penta
                       _|_ Z3β 528      690
                        O      C2       C3
                        |
                       Fuc

D. m/z 852    Fuc-O-Hex-O-|-4HexNAc-O-|-Hex-O-|-4Hex              H type 2 penta
                         325           528      690
                         C2            C3       C4

E. m/z 909    HexNAc-O-HexNAc-O-|-4Hex-O-|-4Hex-O-|-4Hex          Forssman
                                 423      585      747
                                 C2       C3       C4

F. m/z 909    HexNAc-O-Hex-O-|-4HexNAc-O-|-Hex-O-|-4Hex           x2 penta
                             382            585     747
                             C2             C3      C4

G. m/z 998    Fuc-O-Hex-|-O-4HexNAc-O-Hex-O-|-4Hex                Ley hexa
                       325      _|_ Z3β     836
                       C2α       O          C4
                                 |
                                Fuc

H. m/z 1055    HexNAc-O-Hex-O-|-4HexNAc-O-|-Hex-O-|-4Hex          A type 2 hexa
                         |    528           731      893
                         O    C2            C3       C4
                         |
                        Fuc

I. m/z 1201    HexNAc-O-Hex-O-|-4HexNAc-O-|-Hex-O-|-4Hex          A type 2 hepta
                         |    528    3      877      1039
                         O    C2α   _|_Z3β  C3       C4
                         |           |
                        Fuc          O
                                     |
                                    Fuc
```

Supplementary Figure S2. Interpretation formulas for Figure 3 and Figure 5.

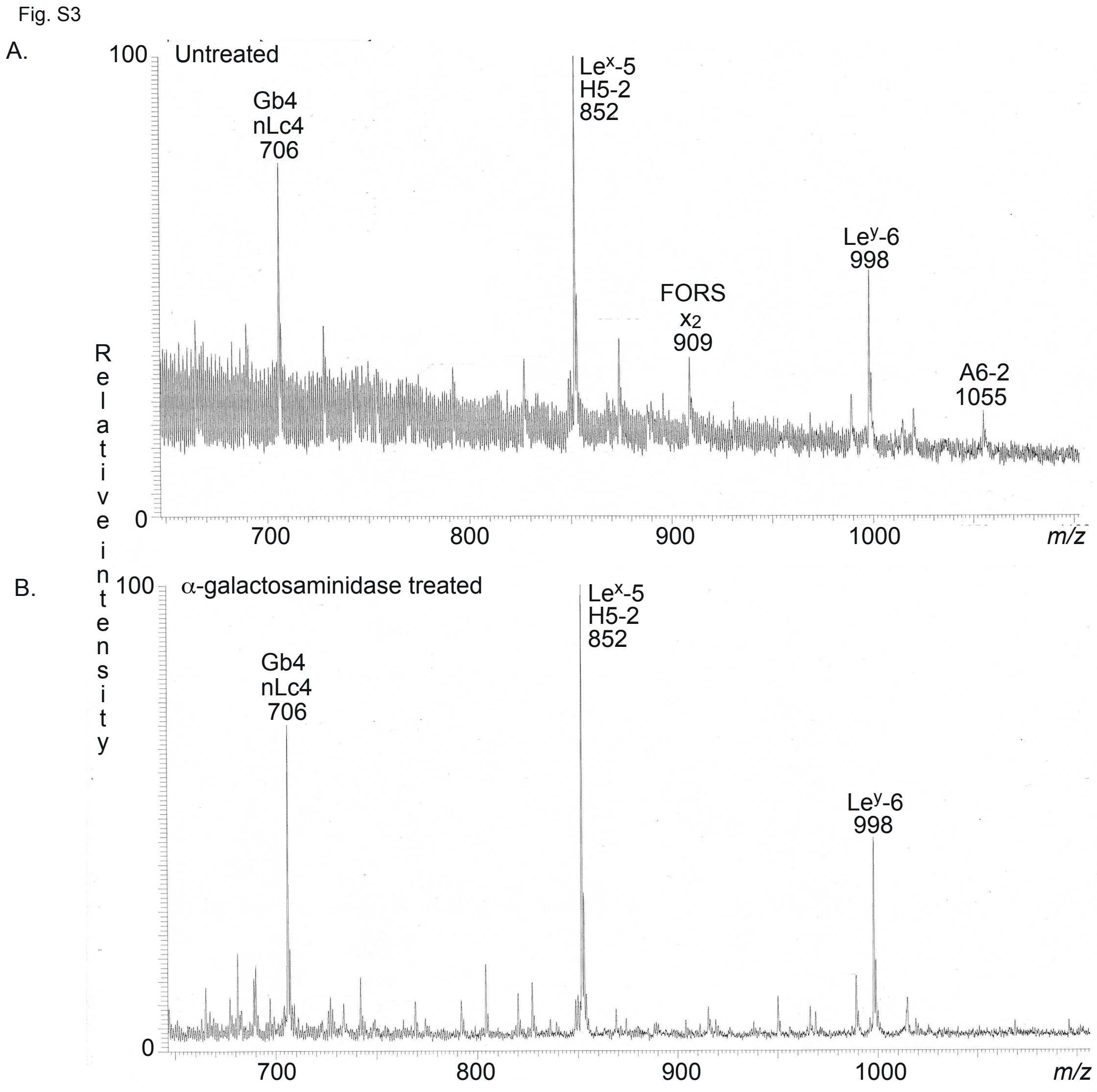

Supplementary Figure S3. α-*N*-acetylgalactosaminidase hydrolysis of the oligosaccharides derived from the total non-acid glycosphingolipid fraction from human medullary thyroid cancer by hydrolysis with endoglycoceramidase II from *Rhodococcus* spp. (A) Molecular ion profile from LC-ESI/MS of the untreated oligosaccharides. (B) Molecular ion profile from LC-ESI/MS of the oligosaccharides after α-galactosaminidase hydrolysis.

The oligosaccharides identified in the chromatogram were: Gb4, GalNAcβ3Galα4Galβ4Glc; nLc4, Galβ4GlcNAcβ3Galβ4Glc; Le^x-5, Galβ4(Fucα3)GlcNAcβ3Galβ4Glc; H5-2, Fucα2Galβ4GlcNAcβ3Galβ4Glc; FORS, GalNAcα3GalNAcβ3Galα4Galβ4Glc; x_2, GalNAcβ3Galβ4GlcNAcβ3Galβ4Glc; Le^y-6, Fucα2Galβ4(Fucα3)GlcNAcβ3Galβ4Glc.

Fig. S4

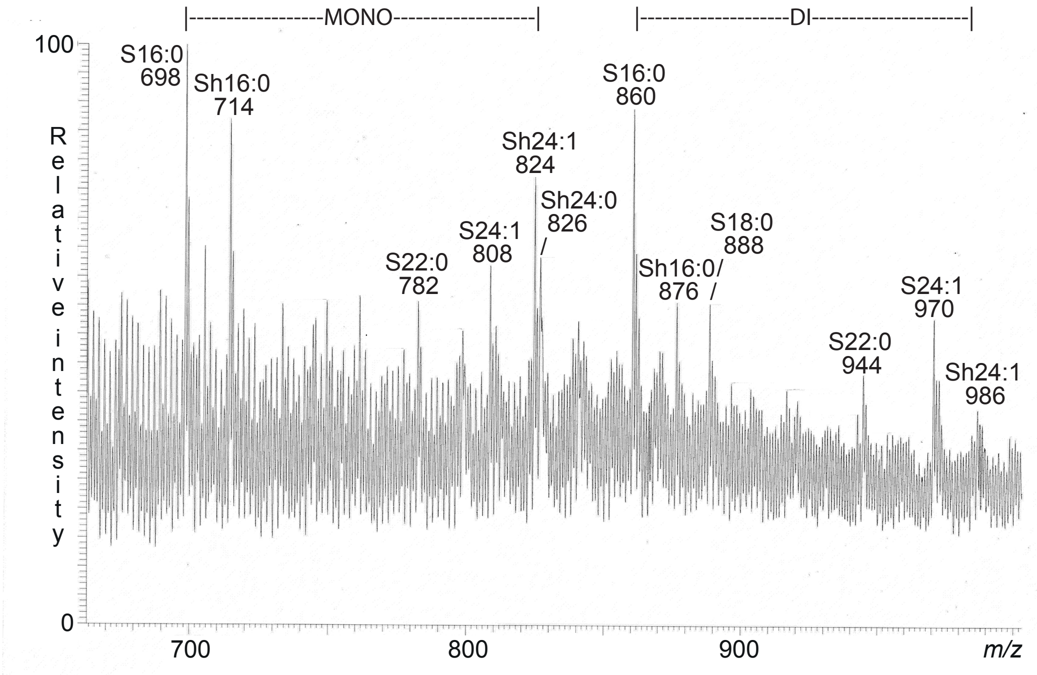

Supplementary Figure S4. Molecular ion profile from LC-ESI/MS of fraction T1. In the shorthand nomenclature for fatty acids and bases, the number before the colon refers to the carbon chain length and the number after the colon gives the total number of double bonds in the molecule. Fatty acids with a 2-hydroxy group are denoted by the prefix h before the abbreviation, as *e.g.* h16:0. S designates sphingosine (d18:1) long chain base.

Supplementary tables

Supplementary Table S1. Glycosphingolipid preparations

	Dry weight	Total acid glycosphingolipids	Total non-acid glycosphingolipids	mg acid glyco-sphingolipids/g dry weight	mg non-acid glyco-sphingolipids/g dry weight
Medullary thyroid cancer	5.4 g	84.4 mg	24.4 mg	15.6	4.5

Supplementary Table S2. Carbohydrate binding ligands used in chromatogram binding assays.

**Ligand**	**Clone**	**Manufacturer**	**Dilution**	**Binding specificity**
Anti-sialyl-Le[a]	116-NS-19-9	Thermo Fischer	1:30	Neu5Acα3Galβ3(Fucα4)GlcNAc
Anti-GD1a	GD1a-1	Merck Millipore;	1:100	Neu5Acα3Galβ3GalNAcβ4(Neu5Acα3)Galβ4Glc
H. pomatia lectin	-	Sigma-Aldrich	-	GalNAcα
Anti-blood group A	HE-195	Sigma-Aldrich	1:500	GalNAcα3(Fucα2)Gal
Anti-blood group H type 2	A583	DakoCytomation Norden A/S	1:100	Fucα2Galβ4GlcNAc
Anti-Lewis[X]	P12	Merck Millipore	1:100	Galβ4(Fucα3)GlcNAc
Anti-Lewis[y]	F3	Merck Millipore	1:100	Fucα2Galβ4(Fucα3)GlcNAc
Anti-Forssman	M1/87	Santa Cruz	1:100	GalNAcα3GalNAcβ3Galα4Galβ4Glc
Anti-GloboH	VK9	eBioscience/ Invitrogen	1:100	Fucα2Galβ3GalNAcβ3Galα4Galβ4Glc

Supplementary Table S3. Characteristics of patients studied by immunohistochemistry

Patient No.	Number of sections	Blood group	Tumor type	Hereditary
1	2	A(Rh+)	Primary tumor	MEN2A[a]
2	4	O(Rh+)	Lymf node metastasis	-
3	1	B(Rh+)	Lymf node metastasis	MEN2B[b]
4	2	O(Rh+)	Primary tumor + Lymf node metastasis	-
5	1	A(Rh+)	Primary tumor	-

[a]Multiple neuroendocrine neoplasia type 2A
[b]Multiple neuroendocrine neoplasia type 2B
